# Capecitabine in combination with irinotecan (XELIRI), administered as a 2-weekly schedule, as first-line chemotherapy for patients with metastatic colorectal cancer: a phase II study of the Spanish GOTI group

**DOI:** 10.1038/sj.bjc.6605261

**Published:** 2009-09-08

**Authors:** P Garcia-Alfonso, A Muñoz-Martin, M Mendez-Ureña, R Quiben-Pereira, E Gonzalez-Flores, G Perez-Manga

**Affiliations:** 1 Medical Oncology Service, Hospital General Universitario Gregorio Marañón, Doctor Esquerdo 46, Madrid 28007, Spain; 2 Medical Oncology Service, Hospital Universitario de Móstoles, Río Júcar s/n, Móstoles, Madrid 28935, Spain; 3 Medical Oncology Service, Hospital Universitario Virgen de las Nieves, Avda. Fuerzas Armadas 2, Granada 18014, Spain

**Keywords:** capecitabine, colorectal cancer, combined modality therapy, irinotecan

## Abstract

**Background:**

Combination chemotherapy is standard treatment for metastatic colorectal cancer (MCRC). The aim of this study was to determine the efficacy and safety of capecitabine+irinotecan (2-weekly schedule), as first-line therapy of MCRC.

**Methods:**

Patients received irinotecan 175 mg m^−2^ on day 1 and oral capecitabine 1000 mg m^−2^ twice daily on days 2–8 every 2 weeks. For patients aged ⩾65 years, the starting doses of irinotecan and capecitabine were reduced to 140 and 750 mg m^−2^, respectively.

**Results:**

A total of 53 patients were enrolled: 29 (55%) were ⩾65 years old. In an intention-to-treat analysis, complete response was achieved in three patients for an overall response rate (ORR) of 32%. The disease control rate (ORR + stable disease) was 66% and the median duration of response was 7.3 months. Median time to progression and overall survival were 9.0 and 19.2 months, respectively. Grade 4 neutropenia was reported in one patient: no other grade 4 toxicities were recorded. Grade 3 diarrhoea occurred in 8 (15%) patients and grade 1–2 hand–foot syndrome in 7 (13%) patients.

**Conclusion:**

Capecitabine and irinotecan, given every 2 weeks, as first-line treatment of MCRC is an active regimen with a manageable toxicity profile, even in older patients.

The addition of irinotecan to infusional (FOLFIRI: every 2 weeks) or bolus (IFL: weekly) 5-fluorouracil/leucovorin (5-FU/LV) has been shown to significantly improve progression-free survival, overall survival (OS), and response rate compared with the equivalent 5-FU/LV regimens alone in the first-line treatment of metastatic colorectal cancer (MCRC) (Douillard *et al*, 2000; Saltz *et al*, 2000). However, FOLFIRI appears to be better tolerated than IFL and is associated with a lower frequency of toxicities such as diarrhoea, myelosuppression, and sepsis (Benson and Goldberg, 2003; Alimonti *et al*, 2004; Hwang, 2004).

Several studies show that patients have a strong preference for oral, rather than intravenous, chemotherapy (Liu *et al*, 1997; Borner *et al*, 2002; Twelves *et al*, 2006): the main reasons for this include convenience, problems with intravenous lines (pain, thrombosis or infection; Mueller *et al*, 1992), and the opportunity to control the environment in which chemotherapy is received. Capecitabine, an inactive oral prodrug, which is preferentially converted to 5-FU in tumour tissue (Meropol, 1998), has emerged as an attractive oral alternative to intravenous 5-FU. Twice-daily oral administration of capecitabine results in continuous exposure to 5-FU, thus avoiding the need for central venous access.

Capecitabine has shown, in large-scale randomised studies in patients with MCRC, a significantly superior response rate and at least equivalent OS compared with bolus 5-FU/LV (Hoff *et al*, 2001; Van Cutsem *et al*, 2001). Moreover, capecitabine showed a superior safety profile with significantly less cases of neutropenia, alopecia, diarrhoea, and stomatitis, but more cases of hand–foot syndrome (Cassidy *et al*, 2002). Following these results, several investigators tested the combination of irinotecan with capecitabine in an attempt to improve on the available 5-FU/LV-based regimens. Encouraging efficacy results from phase II studies were reported with combination regimens of capecitabine and irinotecan given weekly, on days 1 and 8 (CAPIRI) or 3-weekly (XELIRI) as first-line treatment for MCRC (Bajetta *et al*, 2004; Park *et al*, 2004; Borner *et al*, 2005; Cartwright *et al*, 2005; Kim *et al*, 2005; Rea *et al*, 2005; Patt *et al*, 2007). However, dose reductions of both drugs were necessary in most studies to improve the safety profile of these regimens. The 3-weekly XELIRI regimen was selected for further testing in phase III studies. Although the large CAIRO study (Koopman *et al*, 2007) showed that, with careful dose management, the XELIRI regimen was effective and had an acceptable tolerability profile, XELIRI was associated with unacceptable toxicity in two other phase III trials (Fuchs *et al*, 2007; Kohne *et al*, 2008). These data suggest that the optimal doses and schedule for the capecitabine–irinotecan combination have yet to be identified.

The objective of this phase II study was to evaluate the feasibility of a new 2-weekly schedule of capecitabine and irinotecan (similar to the FOLFIRI scheme) in an attempt to improve the tolerability of this combination as first-line therapy in patients with MCRC.

## Materials and methods

Local ethics committee approval was obtained before enrolment of any patient into this multicentre, national, open-label, phase II trial. It was conducted in accordance with the Declaration of Helsinki Principles and its subsequent amendments, as well as Good Clinical Practice Guidelines. Signed informed consent was obtained from all patients before study entry.

The primary study objective was to determine the overall response rate (ORR) to the 2-weekly capecitabine–irinotecan regimen. Secondary objectives included duration of response, time to progression (TTP), OS, and tolerability.

### Eligibility criteria

Patients with histologically confirmed locally advanced colorectal cancer or MCRC, which was measurable according to the RECIST criteria (Therasse *et al*, 2000), were eligible. Patients were either chemotherapy-naive or were to have undergone adjuvant chemotherapy ⩾6 months before study entry. Other eligibility criteria included: age 18–75 years; Eastern Cooperative Oncology Group (ECOG) performance status ⩽2; life expectancy of more than 3 months; adequate haematological, liver, and renal function (i.e., haemoglobin ⩾10 g dl^−1^, absolute neutrophil count of ⩾1.5 × 10^9^ l^−1^, and platelets ⩾100 × 10^9^ l^−1^; total bilirubin ⩽1.25 × the upper limit of normal (ULN), serum transaminase and alkaline phosphatase levels of ⩽2.5 × ULN (total bilirubin ⩽1.5 × ULN, serum transaminase and alkaline phosphatase levels ⩽5 × ULN, in case of liver metastases); creatinine ⩽1.5 × ULN); absence of any primary tumour other than non-melanoma skin cancer or *in situ* cervical carcinoma. Patients with central nervous system metastasis, unresolved bowel obstruction or subobstruction, inflammatory enteropathy, malabsorption syndrome, any uncontrolled chronic disease, or organ allografts were excluded.

### Treatment schedule

On the basis of our previous phase I study (Garcia-Alfonso *et al*, 2003), patients received irinotecan 175 mg m^−2^ as a 30-min intravenous infusion on day 1, followed by oral capecitabine 1000 mg m^−2^ twice daily on days 2–8, every 2 weeks. Taking into consideration the irinotecan package insert suggestions (i.e., caution in patients ⩾65 years and a lower dose in patients ⩾70 years), the starting doses of both irinotecan and capecitabine were reduced to 140 and 750 mg m^−2^ twice daily, respectively, in patients ⩾65 years of age. The study treatment was continued until disease progression, severe toxicity, treatment refusal (unrelated to toxicity), investigator decision, or death.

Both drugs were administered according to the guidelines used for irinotecan monotherapy, including recommendations for the use of concurrent antiemetics, atropine, and loperamide. Appropriate dose interruptions/reductions for capecitabine and/or irinotecan were implemented in the event of specific toxicities, depending on their nature and intensity.

Patients were assessed for toxicity before each infusion using the National Cancer Institute-Common Toxicity Criteria (NCI-CTC, version 2.0) of April 1999.

### Evaluations during the study

Physical examination and laboratory studies, including complete blood counts, serum liver function tests, calcium, ions, and creatinine levels, were performed within 7 days of enrolment. Electrocardiography was carried out and carcinoembryonic antigen levels were determined within 21 days before starting of the treatment. A chest radiograph or computed tomography (CT) scan, and abdominal CT scan were completed within 4 weeks of enrolment. During treatment, safety assessments, biochemical analysis, and blood counts were performed before each cycle (every 2 weeks).

Evaluation of disease status, according to the RECIST criteria (Therasse *et al*, 2000), was carried out every three cycles (6 weeks) during treatment, and every 8 weeks subsequently until disease progression, death, or loss to follow-up.

The duration of response was measured from the first documentation of response to disease progression (PD). TTP was calculated as the time from inclusion in the study until the first report of PD. Patients with no evidence of PD at the time of their last visit were censored at that time. OS was measured from the time of inclusion to date of death.

### Statistical considerations

Sample size was calculated using the optimal method described by Simon (1989). We assumed a minimum efficacy (p0) of 20% and an optimal 40% response rate with the study combination (p1) (similar to the 35–39% obtained with the combination of irinotecan and 5-FU/LV in two pivotal trials; Douillard *et al*, 2000; Saltz *et al*, 2000), with *α*- and *β*-error probabilities of 0.05 and 0.1, respectively. With these considerations, if ⩾16 of a total of 54 patients responded to treatment, further phase III or other trials would be warranted.

All efficacy and safety analyses were carried out on the intention-to-treat (ITT) population, which included all recruited patients. The probabilities of time-to-event parameters were estimated using the Kaplan–Meier method with 95% confidence intervals (95% CIs).

## Results

Between June 2002 and January 2005, a total of 53 patients from three Spanish centres were enrolled into the study. The baseline characteristics of the patients are summarised in Table 1. Of these, 47 (89%) patients had an ECOG performance status of <2 at baseline, 29 (55%) were at least 65 years old, and 57% had at least two organs involved (median 2 and maximum 8), with the liver being the most common site of metastatic disease.

### Treatment compliance

A total of 489 cycles were administered with a median of 11 (range 2–12) cycles per patient. Irinotecan doses were delayed in 56 cycles (12%), and dose reductions were required in 33 cycles (7%). Globally, 3423 doses of capecitabine were administered: of those, 106 administrations (3.1%) were delayed, and dose reductions were required in 171 cases (5%). No significant differences in drug administrations were observed between patients aged >65 and ⩽65 years.

### Toxicity

All patients were evaluable for toxicity. The main haematological and non-haematological toxicities by patient are summarised in Table 2. Grade 4 neutropenia was reported in one patient; no other grade 4 toxicities were recorded. The most frequent non-haematological toxicities were gastrointestinal events (i.e. nausea, vomiting, and diarrhoea), as well as alopecia and asthenia; in most cases, these toxicities did not reach grade >2. In particular, grade 3 diarrhoea was uncommon (eight patients, 11 cycles), and grade 1–2 hand–foot syndrome was observed in seven patients (no grade >2 hand–foot syndrome was reported).

No significant differences related to toxicity were observed in patients aged ⩾65 years and those <65 years, although there was a tendency for a later recovery in older patients (data not shown).

There were no treatment-related deaths during the study or within 28 days after the last study dose.

### Efficacy assessment

In an ITT analysis, in which all 53 patients were considered, 3 (6%) patients attained a complete response and 14 patients a partial response, for an ORR of 32% (95% CI: 20–46%). In addition, 18 (34%) patients had stable disease, providing a disease control rate (ORR + stable disease) of 66% (95% CI: 52–79%). The median duration of response was 7.3 (95% CI: 6.2–8.8) months.

The median TTP and OS were 9.0 (95% CI: 5.7–10.4) and 19.2 (95% CI: 14.1–23.3) months, respectively.

## Discussion

Combination chemotherapy is standard treatment for MCRC, often combined with a biological agent. Until recently oxaliplatin regimens have probably been the most used as first line treatment of MCRC, but as oxaliplatin adjuvant therapy is now usually selected, irinotecan combinations are more likely to be used at first progression. Combining irinotecan with capecitabine will make a regimen that is better tolerated and preferred by patients.

This phase II study shows that a novel 2-weekly schedule of capecitabine plus irinotecan as first-line treatment for MCRC is feasible, with notable efficacy and safety, even in older patients. To date, we are not aware of the publication of any other phase II prospective study of capecitabine plus irinotecan using this schedule. The choice of this regimen was based on our previous phase I study (Garcia-Alfonso *et al*, 2003), which aimed to simulate the FOLFIRI scheme, thereby replacing 5-FU/LV with oral capecitabine.

Previous phase II trial results suggested that the 3-weekly XELIRI regimen was less toxic than those involving irinotecan given on days 1 and 8 (CAPIRI) or on a weekly basis (Bajetta *et al*, 2004; Borner *et al*, 2005), but the initial doses used with 3-weekly XELIRI in those trials had to be reduced in a considerable number of patients. Moreover, XELIRI showed better efficacy than the weekly regimen (Borner *et al*, 2005), probably because of the higher dose intensity of irinotecan in the XELIRI arm. Thus, the 3-weekly XELIRI regimen was taken forward for phase III testing. Although XELIRI was found to be effective with a manageable toxicity profile in the large CAIRO trial (Koopman *et al*, 2007), unacceptable rates of toxicity were reported in both the EORTC 40015 (Kohne *et al*, 2008) and BICC-C (Fuchs *et al*, 2007) studies, both of which compared XELIRI with 5-FU-based irinotecan combinations with a second randomisation to placebo or celecoxib. These findings suggested that the optimal capecitabine–irinotecan schedule had not yet been identified.

Although a comparison of results from different phase II studies can be only speculative, the efficacy of our schedule is in line with that obtained by the other groups who have also published response data in phase II trials testing capecitabine–irinotecan combination regimens (Bajetta *et al*, 2004; Park *et al*, 2004; Borner *et al*, 2005; Cartwright *et al*, 2005; Kim *et al*, 2005; Rea *et al*, 2005; Patt *et al*, 2007; Choi *et al*, 2008) (Table 3). Indeed, our median TTP and OS are among the highest of those published to date with this combination. Moreover, the time-to-event results in this study compare favourably with those reported from recent phase III trials of the FOLFIRI regimen (Tournigand *et al*, 2004; Van Cutsem *et al*, 2007): median TTP (9.0 *vs* progression-free survival of 8.0 and 8.5 months) and median OS (19.2 *vs* 21.5 months).

It is noteworthy that the regimen tested in this study shows good tolerability (dose reductions were required in only 7% of cycles), with predominantly mild or moderate adverse events only. Importantly, there were no treatment-related deaths during our study. No grade 4 non-haematological toxicities were reported and only one case (3%) of non-complicated grade 4 neutropenia was documented. As expected with this combination, diarrhoea was the main adverse event, but with a low rate of grade 3 events (15% of patients) and no grade 4 events. Hand–foot syndrome, a well-documented capecitabine-associated event, was only reported in seven patients and all cases were grade 1 or 2. In Table 3, which summarises the main grade 3–4 toxicities per patient reported in previous phase II studies testing the combination of capecitabine and irinotecan, the improved tolerability of the 2-weekly regimen is notable: grade 3–4 neutropenia and diarrhoea ranged between 5–28 and 8–34% in other phase II studies, respectively, compared with respective rates of 6 and 15% in this study. Unlike the current study, grade 3–4 hand–foot syndrome was reported in most of those studies (Cartwright *et al*, 2005; Patt *et al*, 2007; Choi *et al*, 2008). Our regimen also appears to be associated with a lower rate of grade 3–4 neutropenia (6 *vs* 24–23%) and a similar rate of grade 3–4 diarrhoea (15 *vs* 14–11%) compared with FOLFIRI (Tournigand *et al*, 2004; Van Cutsem *et al*, 2007).

Implementation of up-front dose reductions in older patients also appears to have contributed to a better tolerability profile than has been previously reported for other capecitabine–irinotecan regimens. This approach has also been used successfully with the XELIRI regimen in patients with other known risk factors, for example, renal impairment, previous pelvic irradiation, and older age (Patt *et al*, 2007; Van Cutsem *et al*, 2007).

In conclusion, 2-weekly administration of irinotecan in combination with capecitabine is effective and safe as first-line chemotherapy for advanced colorectal cancer or MCRC and may be suitable for use with targeted agents, such as cetuximab or bevacizumab. Therefore, phase III studies of this regimen are warranted.

## Figures and Tables

**Table 1:** Patient characteristics (*n*=53)

**Parameter**	**No. of patients**	**%**
*Sex*		
Male	39	74
Female	14	26
		
*Age, years*		
Median	66	
Range	42–80	
		
*ECOG performance status*		
0	17	32
1	30	57
2	3	6
Non-specified	3	6
		
*Primary site*		
Colon	30	57
Rectum	22	41
Both	1	2
		
*Metastatic site*		
Liver	31	61
Lung	13	26
Peritoneum	8	16
Other	15	29
		
*Prior therapy*		
Radiotherapy	10	19
Surgery	41	77
Adjuvant chemotherapy	22	42

Abbreviation: ECOG=Eastern Cooperative Oncology Group.

**Table 2:** Maximum toxicity per patient according to the NCI-CTC grade (*n*=53)

	**All grades**		**Grade 3**	
	***n***	**%**	***n***	**%**
*Haematological events*				
Anaemia	23	43	—	—
Leucopenia	4	8	—	—
Neutropenia	12	23	3	6
Thrombopenia	3	6	1	2
				
*Non-haematological events*				
Fever	8	15	—	—
Alopecia	14	26	2	4
Nausea	30	57	4	8
Vomiting	24	45	2	4
Diarrhoea	27	51	8	15
Epigastralgia	8	15	1	2
Asthenia	30	57	7	13
Headache	3	6	—	—
Hand–foot syndrome	7	13	—	—
Mucositis	11	21	—	—
Liver	2	4	—	—
Renal	2	4	1	2
Skin	3	6	—	—
Anorexia	11	21	1	2

Abbreviation: NCI-CTC=National Cancer Institute-Common Toxicity Criteria.

**Table 3:** Phase II studies of capecitabine + irinotecan as first-line treatment of metastatic colorectal cancer

					**Grade 3/4 events (%)**	
**Schedule**	***N***	**RR (%)**	**Median TTP (months)**	**Median OS (months)**	**Neutropenia**	**Diarrhoea**
XELIRI (Bajetta *et al*, 2004)	140	47^a^	8.3^b^	—	5	36
CAPIRI		44^a^	7.6^b^			17
XELIRI weekly (Borner *et al*, 2005)	75	34	6.9	17.4	5	34
XELIRI		35	9.2	24.7	19	19
XELIRI Park *et al*, 2004)	39	44	6.7	—	10	10
XELIRI (Patt *et al*, 2007)	52	50	7.8	16.8	25	20
XELIRI (Rea *et al*, 2005)	57	42	8.3	—	28	19
XELIRI (Cartwright *et al*, 2005)	49	45^a^	6.2^b^	13.4	12	20
CAPIRI (Kim *et al*, 2005)	47	49	7.5	19.5	11	24
XELIRI 2-weekly (Choi *et al*, 2008)	43	51	10.0	15.0	5	8
XELIRI 2-weekly (this study)	53	32	9.0	19.2	6	15

Abbreviations: CAPIRI=capecitabine on days 2–15 + irinotecan on days 1 and 8; OS=overall survival; RR=response rate; TTP=time to progression; XELIRI=capecitabine on days 1–14 + irinotecan on day 1 every 3 weeks; XELIRI weekly=capecitabine on days 1–14 + irinotecan on days 1, 8 and 15 every 3 weeks.

a RR in evaluable patients.

b Progression-free survival.
